# Supplementary material for: An Evaluation of Laminarin Additive in the Diets of Juvenile Largemouth Bass (Micropterus salmoides): Growth, Antioxidant Capacity, Immune Response and Intestinal Microbiota
Source: Animals (Basel). 2023 Jan 28;13(3):459. doi: 10.3390/ani13030459 (PMC9913627; doi:10.3390/ani13030459)
Supplement: Supplementary file 1 [file animals-13-00459-s001.zip › Table S1.pdf]

Table S1 Raw reads and clean reads of 16 sRNA sequence

Sample ID	Raw Reads	Clean Reads	Denoised Reads	Merged Reads	Non-chimeric Reads
Con1	66515	66430	65571	61885	56437
Con2	119527	119090	117788	113086	106847
Con3	44872	44808	44135	41141	37515
Con4	80011	79910	79060	75525	69442
HL1	71167	70886	69317	68594	67321
HL2	74220	74011	70841	70024	68381
HL3	100054	99671	94797	94180	92289
HL4	94719	94291	93388	92282	90979
LL1	50471	50397	37445	33427	31271
LL2	79888	79800	57344	51351	49217
LL3	80050	79957	57627	51147	48213
LL4	45528	45472	33742	29878	28485
