# Supplementary material for: An Evaluation of Laminarin Additive in the Diets of Juvenile Largemouth Bass (Micropterus salmoides): Growth, Antioxidant Capacity, Immune Response and Intestinal Microbiota
Source: Animals (Basel). 2023 Jan 28;13(3):459. doi: 10.3390/ani13030459 (PMC9913627; doi:10.3390/ani13030459)
Supplement: Supplementary file 1 [file animals-13-00459-s001.zip › Table S2.pdf]

Table S2 The Firmicutes/Bacteroidetes ratio

	Con	LL	ML	HL
Firmicutes	0.143525381	0.332473935	0.086984506	0.064129049
Bacteroidota	0.102547391	0.083171084	0.098994172	0.003366488
Firmicutes/Bacteroidetes ratio	1.399600508	3.997470266	0.878683098	19.04924242
